# Editorial: Evolution in cardiovascular MedTech

**DOI:** 10.3389/fmedt.2026.1812982

**Published:** 2026-03-24

**Authors:** Mauro Malvè, Rosaire Mongrain, Stephane Avril

**Affiliations:** 1 Public University of Navarre, Pamplona, Spain; 2 Centro de Investigacion Biomedica en red en Bioingenieria Biomateriales y Nanomedicina, Zaragoza, Spain; 3 Department of Mechanical Engineering, McGill University, Montreal, QC, Canada; 4 Ecole des Mines de Saint-Etienne, Saint-Étienne, France; 5 SAnte INgenierie BIOlogie St-Etienne, St-Priest-en-Jarez, France

**Keywords:** auxetic stent structures, cardiovascular intervention innovation, computational hemodynamics, coronary bifurcation stenting techniques, finite element modeling, magnetocardiography, neuromuscular electrical stimulation therapy, thrombectomy catheter designs

Over the past decades, cardiovascular medicine has undergone an important transformation driven by technological innovation, computational modelling, and an expanding understanding of biological mechanisms. Engineering advances have enabled clinicians and researchers to tackle long standing challenges in diagnosis, device design, procedural planning, and therapeutic effectiveness. The Research Topic Evolution in Cardiovascular MedTech brings together an accurate set of studies that exemplify this development, blending foundational research, translational modelling, device innovation, and clinical insights. Together, the eight contributions in this collection offer a snapshot of the current landscape, where interdisciplinary approaches are redefining what is possible in cardiovascular care.

The first set of articles, published in Frontiers in Medical Technology, addresses both computational and experimental frameworks that seek to improve risk assessment, device design, and mechanistic understanding in cardiovascular interventions. Astori et al. open the topic with a study that tackles a critical clinical problem in congenital and pediatric care: risk assessment of coronary artery compression in pulmonary conduit pre stenting. By employing finite element analysis, the authors explore the interplay between modelling complexity, computational expense, and predictive reliability. Their work highlights how patient specific simulations can support procedural planning by balancing accuracy with practical resource constraints—a theme that resonates across many domains of cardiovascular technology, where personalised risk assessment is becoming increasingly important.

Building on the theme of diagnostic innovation, He et al. investigate the utility of magnetocardiography (MCG) as a non-invasive tool for functional assessment of myocardial ischemia in patients with stable coronary artery disease. Through rigorous validation against established standards, this study shows that MCG may offer additional functional insight beyond conventional electrocardiography, particularly in subtle cases of ischemic burden. Such advances in non-invasive functional assessment underscore the growing role of novel sensing modalities in cardiovascular diagnostics, particularly when combined with sophisticated signal processing.

In the domain of interventional cardiology, Renon et al. explore how computational modelling can inform the evolving practice of percutaneous coronary intervention with drug coated balloons. Through detailed simulations, the authors illustrate how computational fluid dynamics and structural modelling can provide mechanistic insight into dose distribution, drug transport, and vessel wall response—factors that are difficult to resolve empirically. Importantly, this work reinforces the notion that computational tools are not merely research artefacts but have genuine translational potential to inform device selection, procedural strategy, and optimisation of therapies.

Device design extends beyond simulation alone. Kortman et al. contribute a comprehensive patent review of advancements in aspiration catheter tip design for thrombectomy. By tracking innovation through patent literature, the authors identify key design trends—such as improvements in tip geometry, material selection, and flow steering—that have the potential to influence procedural success and patient outcomes. This sweeping review bridges clinical need and engineering creativity, illustrating how intellectual property trends can provide a leading indicator of future practice.

Experimentation remains crucial for device evaluation. Vellaparambil et al. present an experimental validation of auxetic stent designs using three point bending tests on 3D printed titanium prototypes. Auxetic structures, which exhibit a negative Poisson's ratio, have emerged as promising candidates for cardiovascular scaffolds, offering enhanced conformability and mechanical performance. By combining additive manufacturing with mechanical testing, this study lays the groundwork for future optimisation of stent mechanics—particularly in anatomically challenging locations such as bifurcations or tortuous vessels.

Vento et al. offer a broader vantage point in their perspective article on evolutionary trends and innovations in cardiovascular intervention. By synthesising historical developments and future directions, they articulate how incremental improvements and paradigm shifts alike have shaped contemporary practices. Their analysis contextualises the other contributions in this volume, reminding readers that evolution in medical technology is iterative and multidimensional, spanning materials science, computational modelling, imaging, and procedural innovation.

The final two articles of this Research Topic, published in Frontiers in Cardiovascular Medicine, bridge the technological insights of Medical Technology with clinical outcomes and practical implications. Mochizuki et al. present a case report on the use of belt electrode skeletal muscle electrical stimulation for acute heart failure in a patient with severe obesity. This innovative application highlights how bioelectronic stimulation can serve as a therapeutic option in acute phases and suggests new avenues for rehabilitation strategies, particularly in populations where conventional exercise therapy is limited.

Rounding out the collection, Tu et al. provide valuable long term comparative data on two stenting strategies for true coronary bifurcation lesions: Double Kissing Mini culotte vs. Mini culotte stenting using drug eluting stents. With five-year clinical outcomes, this study contributes much needed evidence on procedural durability, restenosis rates, and adverse event profiles—outcomes that are critically relevant to interventional cardiologists and device developers alike. By anchoring device design and procedural planning in extended clinical follow up, this work exemplifies the type of translational research that connects engineering innovation with real world patient benefit.

Taken together, the articles in Evolution in Cardiovascular MedTech reflect the current state of an interdisciplinary field that is advancing through synergy between engineering and clinical science. Computational models are increasingly used not only for hypothesis generation but also for personalised risk identification; experimental prototypes are being validated with modern manufacturing techniques; and long-term clinical evidence is being systematically integrated with device and procedural innovation.

As the pace of progress accelerates, several cross-cutting themes emerge from this volume. First, the importance of personalisation—whether through patient specific simulations, tailored device geometries, or individualised therapeutic strategies—is becoming a trademark of cardiovascular medtech research. Second, non-invasive diagnostics and advanced sensing modalities are gaining traction as complements or alternatives to traditional methods. Third, the integration of computational, experimental, and clinical data is crucial for addressing complex pathophysiological questions and for translating insights into better care.

We thank all the authors for their contributions to this Research Topic, and we hope that this collection stimulates further research that continues to bridge disciplinary boundaries. The challenges of cardiovascular disease are multifaceted, but so too are the tools at our disposal. By fostering collaboration between engineers, clinicians, and scientists, the research in this field will benefit patients and advance our collective understanding of cardiovascular health.

